# Antimicrobial Analysis of an Antiseptic Made from Ethanol Crude Extracts of *P. granatum* and *E. uniflora* in *Wistar* Rats against *Staphylococcus aureus* and *Staphylococcus epidermidis*

**DOI:** 10.1155/2015/751791

**Published:** 2015-06-03

**Authors:** Thaís Honório Lins Bernardo, Regina Célia Sales Santos Veríssimo, Valter Alvino, Maria Gabriella Silva Araujo, Raíssa Fernanda Evangelista Pires dos Santos, Max Denisson Maurício Viana, Maria Lysete de Assis Bastos, Magna Suzana Alexandre-Moreira, João Xavier de Araújo-Júnior

**Affiliations:** ^1^The Northeast Biotechnology Network (RENORBIO), Brazil; ^2^School of Nursing and Pharmacy (ESENFAR), Campus A.C. Simões, Avenue Lourival Melo Mota, 101 North Km 97, Tabuleiro dos Martins, 57072-970 Maceió, AL, Brazil; ^3^Institute of Health Sciences, Federal University of Alagoas (UFAL), Brazil

## Abstract

*Introduction*. Surgical site infection remains a challenge for hospital infection control, especially when it relates to skin antisepsis in the surgical site. *Objective*. To analyze the antimicrobial activity *in vivo* of an antiseptic from ethanol crude extracts of *P. granatum* and *E. uniflora* against Gram-positive and Gram-negative bacteria. *Methods*. Agar drilling and minimal inhibitory tests were conducted for *in vitro* evaluation. In the *in vivo* bioassay were used *Wistar* rats and *Staphylococcus aureus* (ATCC 25923) and *Staphylococcus epidermidis* (ATCC 14990). Statistical analysis was performed through variance analysis and Scott-Knott cluster test at 5% probability and significance level. *Results*. In the *in vitro*, ethanolic extracts of *Punica granatum* and *Eugenia uniflora* and their combination showed the best antimicrobial potential against *S. epidermidis* and *S. aureus*. In the *in vivo* bioassay against *S. epidermidis*, there was no statistically significant difference between the tested product and the patterns used after five minutes of applying the product. *Conclusion*. The results indicate that the originated product is an antiseptic alternative source against *S. epidermidis* compared to chlorhexidine gluconate. It is suggested that further researches are to be conducted in different concentrations of the test product, evaluating its effectiveness and operational costs.

## 1. Introduction

The prevention of SSIs is based on knowledge of various risk factors that predispose the patient to develop an infection and, also, on the comprehension of microbiology [1].

Prevention interventions can be organized addressing the preoperative risk factors of the patient and those related to perioperative management, including the preparation of the surgical team, the environment where the surgery will be performed, the techniques used during the procedure, and intraoperative and postoperative care [1].

Proper skin preparation in the preoperative period is a well-established preventive measure against SSIs. The preparation is performed by antisepsis, a simple and effective measure for the prevention of infections, and is essential to health care preceding a surgery procedure [1, 2].

Several antiseptic agents are available for preoperative skin preparation the incision site. The antiseptic chosen must have fast acting, broad spectrum and must be able to suppress the resurgence of the remaining microorganisms during surgery. The most used substances for skin antisepsis are alcohols (ethanol, isopropanol, and n-propanol), chlorhexidine, commonly available as chlorhexidine gluconate (CHG), and povidone-iodine (PVI), an organic complex of iodine [2, 3].

As a means of reducing the onset of SSIs, there was growing interest among researchers in order to search untested substances which have antibacterial action and can act as an antiseptic in preparation of the skin before surgery.

Among the surveyed substances are medicinal plants which have been used since the existence of human civilization by a large proportion of the population living in rural and urban areas for various therapeutical purposes. They represent potentially important sources of new pharmaceutical substances [4, 5].

The reduction in the incidence of infection in surgical patients can cause great benefits such as patient comfort and economy resources. The necessary precautions to reduce hospital infection are of fundamental importance, requiring measures to prevent infection involving both the patient as well as the surgical team [6].

Brazil has a wide variety of plants and therefore has great potential for the development of herbal medicines [7]. The plant species* Punica granatum* L. is a tree about 3 meters high, rich in tannins, essential oils, and phenols [8]. It has been widely used for various therapeutic purposes; recent studies indicate the activity of this plant species as in the case of the research that determined the antibacterial and antifungal activities, where the ethanolic extract showed activity against* Staphylococcus aureus* and* Candida albicans* [9].

*Eugenia uniflora* L., popularly known as Pitanga, is also widely used by the population as an alternative in the treatment of intestinal, inflammatory, and diuretic disorders, among others. In the study performed by Samy et al. (2014), isolated parts of this plant species showed antibacterial activity against* Staphylococcus aureus* strain by the broth dilution method with a minimum inhibitory concentration ranging from 36.9 to 81.9 *μ*M [10].

The plant species* S. siamea* is a medicinal plant used empirically in treating abdominal pain, typhoid, and genitourinary disorders, among others. It consists of saponins, tannins, resins, and steroids. The aqueous extract of the leaves was shown to be effective against infections caused by* Pseudomonas aeruginosa* [11], while the extract of the flowers of this species was active against strains of* Staphylococcus aureus* [12].

*Schinus terebinthifolius* Raddi, popularly known as Aroeira is often found in northeastern Brazil and used to treat respiratory infections. Their structures consist of triterpenic alcohols, ketones, acids, monoterpenes, and sesquiterpenes [13]. The antimicrobial activity of extracts of this species was evaluated in previous studies, which determined the activity against the strains of* Staphylococcus aureus*,* Escherichia coli*,* Pseudomonas aeruginosa,* and* Candida albicans* [14, 15].

## 2. Objective

- Our objective was to evaluate the* in vitro* antimicrobial activity of ethanol crude extracts obtained from plant species* Punica granatum*,* Senna siamea*,* Schinus terebinthifolia* Raddi, and* Eugenia uniflora* against Gram-positive and Gram-negative bacteria.
- Our objective was to analyze the antimicrobial activity* in vivo* of an antiseptic from ethanol crude extracts of* P. granatum* and* E. uniflora*.

## 3. Material and Methods

Leaves samples were collected from the following plants:* Punica granatum*,* Senna siamea*,* Schinus terebinthifolia* Raddi, and* Eugenia uniflora* in Alagoas, Brazil. These plants were selected because they present antimicrobial activity in the literature [16–19].* In vitro* antimicrobial activity was the parameter used for the selection of these plants, because it is an unpublished study, which explores, for the first time, the antiseptic activity of these plant species* in vivo*.

A study found that the quality of antiseptics and disinfectants emphasizes that known* in vitro* antimicrobial activity of antiseptics and disinfectants standard use in health services is of great importance to adjust and rationalize their use to reality. On this basis this research initially sought to determine* in vitro* antimicrobial activity using as positive control the antibiotic ceftriaxone and then test the bioactive* in vivo* as an antiseptic, with reference to the antiseptic chlorhexidine [20].

Samples of these assessed species were duly recognized by the Botanical Institute of the Environment of the State of Alagoas (IMA). The herbarium specimens are cataloged in the herbarium of the IMA with registration number MAC* Punica granatum* (10290),* Senna siamea* (46994),* Schinus terebinthifolia* Raddi (11233), and* Eugenia uniflora* (26078).

The crude extracts were obtained by cold maceration process with cold ethanol (EtOH) at 96° INPM and concentrated by rotary evaporator at 40°C and kept in an incubator, at the same temperature. The solubilization of the crude extracts was performed in saline solution at 0.9% and dimethyl sulfoxide (maximum concentration at 2%). The final concentration of the extract was 2 mg/mL.

Bacterial strains used in this study are standardized by the American Type Cell Collection (ATCC/Manassas, VA/USA):* Staphylococcus aureus* (25923),* Staphylococcus epidermidis* (14990),* Pseudomonas aeruginosa* (27853), and* Escherichia coli* (14942).

Antimicrobial* in vitro* assays were performed by agar drilling method with sterile medium Ágar Mueller-Hinton. The bacterial inoculum was prepared according to the* McFarland* 0.5 scale, corresponding to 1.5 × 10^8^ CFU/mL. The ceftriaxone antibiotic was used as a positive control. The negative control was made by solubilization of the extract solubilization solution DMSO in saline solution at 0.9% (20 *μ*L/mL). The plates were incubated at 36°C for 24 h.

In order to determine the minimum inhibitory concentration (MIC), the inoculum was prepared in standard concentration (10^4^ CFU/mL). The MIC was performed in triplicate as described by CLSI [21] performed in sterile 96-well microplates. The plates were incubated at 36°C for 18 h. After this time, 20 *μ*L of 2,3,5-triphenyl tetrazolium chloride (TTC) at 5% (v/v) in each well was added and the plates were reincubated for 3 hours. Chlorhexidine gluconate was used as a positive control.

Activity level was determined by the following criteria: active with MICs ≤ 100 mg/mL; moderately active 100 < MIC ≤ 500 mg/mL; low activity with 500 < MIC ≤ 1000 mg/mL; and inactive with MICs ≥ 1000 mg/mL [22]. Based on* in vitro* results, final product was formulated from the combination of the* Punica granatum* and* Eugenia uniflora* extracts by showing better antimicrobial activity.

For* in vivo* testing, the study was approved by the Ethics Committee on Animal Use (CEUA) from Federal University of Alagoas, Brazil, and followed the ethical principles of care and management of laboratory animals (number 021066/2011-45) [23].

*Rattus norvegicus albinus* from* Wistar* lineage (*n* = 16), female, at 2 months of age, weighing 150 to 200 g, were used. They were housed in individual cages and randomly divided into 3 groups of 5 animals each. In all animals belonging to each group, two types of antiseptic solutions (one degerming and other alcoholic) were used to ensure equality between the groups. The negative control was tested based on solution formulation of experimental antiseptic, positive control with degerming chlorhexidine 2% and alcoholic chlorhexidine 0.5% (gold standard) and the experimental group tested the antiseptic made from crude ethanol extract of the leaves of plant species* Punica granatum* and* Eugenia uniflora* degerming 2% (V/V) and antiseptic made from crude ethanolic extract of the leaves of plant species* Punica granatum* and* Eugenia uniflora* at 0.5% (V/V).

The use of such formulations was due to the fact that most commonly used skin preparation solutions are chlorhexidine and iodine combined with isopropyl alcohol compound [24].

For* in vivo* study, the strains used were* Staphylococcus aureus* (25923) and* Staphylococcus epidermidis* (14990). They were grown up in brain heart infusion broth and used at a concentration of 0.5 McFarland (10^8^ CFU/mL). Each inoculum was tested at different times totaling two steps.

Rats were anesthetized by the association of ketamine (50 mg/kg) and xylazine (8 mg/kg) by intramuscular route. After anesthesia, the back of the animal was manually trichotomized for achievement of skin culture with identification of the animal natural microbiota. Subsequently, the microorganism was inoculated in unbroken skin (Time 1) and after 10 minutes (Time 2) skin culture was performed to confirm contamination of the animal.

Thereafter, the antisepsis was carried out according to the group and afterwards the new skin cultures were made after five minutes and 1 h after application of the product to be testing intervals for identifying antibacterial action. All skin cultures were performed by means of a sterile swab soaked in saline solution at 0.9%, which, after collection, was spread on plates containing the culture broth brain heart infusion, which are then placed in an oven at 36°C for 24 hours. These procedures were repeated for three consecutive days for each inoculated microorganism, ending with euthanasia of rats.

The assessment of microorganisms presence occurred by counting the number of colony forming units (CFU) per plate. The detection limit for this method was about 100 CFU/plate. The colonies of cultured bacteria were identified through catalase, DNase, coagulase, and novobiocin tests.

The difference in the average number of colonies before and after antisepsis was then compared among groups.

Statistical analysis was performed by analysis of variance (ANOVA) and the Scott-Knott cluster test at 5% probability. Significance was accepted when *p* < 0.05.

## 4. Results

The ethanolic extracts of* Punica granatum*,* Schinus terebinthifolia* Raddi, and* Eugenia uniflora* showed antibacterial activity for all target microorganisms in agar drilling test, showing inhibition zones between 10 and 20 mm. The ethanolic extract of* Senna siamea* showed antibacterial activity only against strains of* Staphylococcus aureus* and* Escherichia coli*, with inhibition zones of 12 mm and 20 mm, respectively (Table 1).

The MIC determination was performed with the microorganisms most commonly associated with SSIs, which in 77% of all cases are Gram-positive cocci. Among these,* Staphylococcus aureus* and* Staphylococcus epidermidis* are responsible for 49% and 28% of cases, respectively [25] (Table 2). Such samples did not inhibit* Pseudomonas aeruginosa* bacterial growth.

The results of MIC determination showed that ethanolic extracts of the leaves of* Punica granatum* and* Eugenia uniflora* and their combination were considered the best antimicrobial potential, being active against* Staphylococcus epidermidis* and moderately active against the strain of* Staphylococcus aureus* (Tables 2 and 3).

Remaining species and their associations were considered moderately active against the two bacterial strains evaluated, except for* Senna siamea* species that showed low activity against the strain of* S. epidermidis* (Tables 2 and 3).

In the* in vivo* tests, the experimental antiseptic products formulated from the association of extracts of* Punica granatum* and* Eugenia uniflora*, which showed better antimicrobial activity* in vitro*, kept presenting their antimicrobial potential.

When comparing the number of bacterial colonies of* Staphylococcus epidermidis* between positive control and experimental group, it was observed that test product showed very similar results, keeping statistically significant difference just five minutes and nine hours after application on animals skin (Table 4).

In comparison with the amount of bacterial colonies of* Staphylococcus aureus*, antiseptic product experimental group showed an underperformance compared to positive control at all the times observed between experimental group and chlorhexidine gluconate (Table 5).

## 5. Discussion

*Punica granatum* and* Eugenia uniflora* extracts, among the tested extracts, showed better antibacterial activity against the strains most commonly found in the human skin microbiota.

Among various plant extracts evaluated in other studies, ethanolic extracts of* Punica granatum* showed better antimicrobial potential against the strains Gram-positive bacteria (including* Staphylococcus aureus* and* Staphylococcus epidermidis*) [26].

Fruit's peel methanolic extract of* Punica granatum* also inhibited the growth of these same bacteria [27], likewise for front Gram-negative bacteria (*Escherichia coli*,* Proteus mirabilis*, and others) [26].

These works corroborate the present study, since the ethanolic extract of the leaves of* Punica granatum* sensitized all target microorganisms in agar drilling test and was active against* Staphylococcus epidermis* and moderately active against the strain of* Staphylococcus aureus* in the MIC test. It remained with this same antimicrobial action in the extracts association from* Punica granatum* and* Eugenia uniflora*.

Whereas* Eugenia uniflora* species also inhibited the growth of all microorganisms evaluated in agar test drilling as well as being shown to be active in the MIC against* Staphylococcus epidermidis* and moderately active for* Staphylococcus aureus*, in association with* Punica granatum*, it remained active for* Staphylococcus epidermidis* and moderately active in other associations.

*Eugenia uniflora* essential oils showed antimicrobial activity against the Gram-positive bacteria* Streptococcus* spp. and* Staphylococcus epidermidis* [28]. Nevertheless, against Gram-negative strains,* Eugenia uniflora* ethanolic extract showed no inhibition of growth. However, when associated with antibiotic, it demonstrated synergism [29].

This research showed that the* Eugenia uniflora* MIC is consistent with Coutinho et al. (2010) and also had a superior outcome to 1000 *μ*g/mL against strains of* E. coli* from the ethanol extract of the same species [29].

The results of this study confirm the findings of previous research, as demonstrated by significant antimicrobial activity against Gram-positive bacteria favoring investments in associations with other extracts also active on the tested strains.

In the associations of extracts, the majority kept their MIC values when tested alone against Gram-positive bacteria. The best antimicrobial result was the association of* Punica granatum* and* Eugenia uniflora* which proved to be active for* Staphylococcus epidermidis*.

In the* in vivo* bioassay, the antiseptic product formulated from the* Punica granatum* and* Eugenia uniflora* association exhibited a similar result to chlorhexidine against* Staphylococcus epidermidis* demonstrating antiseptic potential. Other products tested compared to chlorhexidine as an antiseptic skin showed no significant difference, such as the polihexanide which was indicated as an alternative to chlorhexidine [30].

Since it is an association formed by crude extracts, studies in order to develop association of extracts of* Punica granatum* and* Eugenia uniflora* with chlorhexidine may be promising.

A recent research which associated chlorhexidine with eucalyptus oil showed greater penetration into the deeper layers of the skin significantly compared to aqueous solutions for this ratifying eucalyptus oil as a strategy for improving chlorhexidine [31].

The association of* Punica granatum* and* Eugenia uniflora* showed up with lower antiseptic activity compared to chlorhexidine when tested* in vivo* against* Staphylococcus aureus*. This may be related to the concentration used in the solution of the association, which was similar to the positive control. However, chlorhexidine is a product made from a pure substance, while the experimental product is an association of crude extracts. Further studies may be developed to isolate substance and/or increase the concentration of the extract solution.

## 6. Conclusion

From presented data,* Punica granatum* and* Eugenia uniflora* association represents a potential antiseptic given the positive results against Gram-positive bacteria of the skin microbiota and may represent a successful alternative in the prevention of surgical site infections. Further studies are necessary to improve this product towards concentrations adjustment, quality control, and/or new associations. Moreover, phytochemicals investments of purification and characterization of the active compounds by high-performance liquid chromatography (HPLC) may represent the most active microbiological testing with drugs prototypes being even further promising.

## Figures and Tables

**Table 1:** Antibacterial activity of ethanolic crude extracts.

Microorganisms	Average of inhibition zones (mm)			
	*Staphylococcus aureus *	*Staphylococcus epidermidis *	*Escherichia coli *	*Pseudomonas aeruginosa *
*Punica granatum *	12	13	10	11
*Sennasiamea *	13	12	12	NI
*Schinus terebinthifolia *Raddi	12	12	20	12
*Eugenia uniflora *	11	13	11	14
Positive control (ceftriaxone)	28	26	27,5	25,5
Negative control	N.I.	N.I.	N.I.	N.I.

Note: mm: millimeter; N.I.: did not inhibit.

**Table 2:** Minimum Inhibitory Concentration (MIC) of the evaluated plant species against the strains of *Staphylococcus aureus* and *Staphylococcus epidermidis*.

Microorganisms	Minimum inhibitory concentration (MIC) (*µ*g/mL)	
	*Staphylococcus aureus *	*Staphylococcus epidermidis *
*Punica granatum (Pu) *	500	31
*Senna siamea(Ss) *	250	667
*Schinus terebinthifolia (St) *	250	125
*Eugenia uniflora (Eu) *	250	52
Positive control (CHL)	122,07	3,81
Negative control	0	0

Note: *µ*g/mL: microgram/milliliter; CHL: degerming chlorhexidine; N.I.: did not inhibit.

**Table 3:** Minimum inhibitory concentration (MIC) of the plant species associations evaluated against strains of *Staphylococcus aureus* and *Staphylococcus epidermidis*.

Microorganisms	Minimum inhibitory concentration (MIC) (*µ*g/mL)	
	*Staphylococcus aureus *	*Staphylococcus epidermidis *
Pu + St	500	125
Pu + Ss	500	125
Pu + Eu	500	31
Ss + St	500	250
Ss + Eu	500	250
St + Eu	500	250
Pu + St + Ss	500	125
Pu + St + Eu	500	125
Pu + Ss + Eu	500	125
Ss + St + Eu	250	500
Positive control (CHL)	122,07	3,81
Negative control	0	0

Note: Pu: *Punica granatum*; Ss: *Senna siameu*; St: *Schinus terebinthifolia Raddi*; Eu: *Eugenia uniflora*; *µ*g/mL: microgram/milliliter; CHL: degerming chlorhexidine.

**Table 4:** Counting of colony forming units of *Staphylococcus epidermidis* which grew up in cultures of skin during nine hours after antisepsis.

Time	Negative control	Positive control	Experimental group
After inoculation of bacteria			
10 minutes	88,00^a^	82,40^a^	95,20^a^

After product application			
5 minutes	55,13^a^	1,06^c^	15,93^b^
1 hour	64,86^a^	0,07^b^	14,33^b^
2 hours	45,06^a^	0,07^c^	0,07^c^
3 hours	35,79^a^	0,13^b^	0,00^b^
4 hours	50,80^a^	6,93^b^	0,00^b^
5 hours	26,86^a^	0,07^b^	0,00^b^
6 hours	39,26^a^	0,07^c^	13,86^b^
7 hours	45,06^a^	0,07^b^	8,80^b^
8 hours	57,40^a^	0,07^b^	3,86^b^
9 hours	53,46^a^	0,13^b^	3,80^b^

Note: means followed by the same letter in the line do not differ by Scott-Knott cluster test at 5% probability.

**Table 5:** Counting of colony forming units of *Staphylococcus aureus* which grew up in cultures of skin during nine hours after antisepsis.

Time	Negative control	Positive control	Experimental group
After inoculation of bacteria			
10 minutes	100^a^	100^a^	100^a^

After product application			
5 minutes	35,13^a^	6,73^b^	54,73^a^
1 hour	21,93^a^	0,00^b^	38,86^a^
2 hours	22,86^a^	0,00^b^	35,40^a^
3 hours	13,93^b^	13,33^b^	36,86^a^
4 hours	10,60^a^	6,67^b^	24,73^a^
5 hours	7,06^b^	3,86^b^	31,20^a^
6 hours	17,13^a^	0,20^b^	18,40^a^
7 hours	7,86^a^	0,4^b^	14,86^a^
8 hours	21,53^a^	4,53^b^	13,40^a^
9 hours	26,73^a^	0,00^b^	13,53^a^

Note: means followed by the same letter in the line do not differ by Scott-Knott cluster test at 5% probability.
